# Unusual Clinical Presentation of Ethylene Glycol Poisoning: Unilateral Facial Nerve Paralysis

**DOI:** 10.1155/2013/460250

**Published:** 2013-11-04

**Authors:** Eray Eroglu, Ismail Kocyigit, Sami Bahcebasi, Aydin Unal, Murat Hayri Sipahioglu, Merva Kocyigit, Bulent Tokgoz, Oktay Oymak

**Affiliations:** ^1^Department of Internal Medicine, Erciyes University Medical School, Melikgazi, 38039 Kayseri, Turkey; ^2^Department of Nephrology, Erciyes University Medical School, Melikgazi, 38039 Kayseri, Turkey; ^3^Department of Neurology, Erciyes University Medical School, Melikgazi, 38039 Kayseri, Turkey

## Abstract

Ethylene glycol (EG) may be consumed accidentally or intentionally, usually in the form of antifreeze products or as an ethanol substitute. EG is metabolized to toxic metabolites. These metabolites cause metabolic acidosis with increased anion gap, renal failure, oxaluria, damage to the central nervous system and cranial nerves, and cardiovascular instability. Early initiation of treatment can reduce the mortality and morbidity but different clinical presentations can cause delayed diagnosis and poor prognosis. Herein, we report a case with the atypical presentation of facial paralysis, hematuria, and kidney failure due to EG poisoning which progressed to end stage renal failure and permanent right peripheral facial nerve palsy.

## 1. Introduction

Ethylene glycol (EG) may be consumed accidentally or intentionally, usually in the form of antifreeze products or as an ethanol substitute. It is converted by alcohol dehydrogenase to active metabolites in the liver, and these metabolites cause metabolic acidosis with increased anion gap, renal failure, hypocalcemia, oxaluria, and damage to the central nervous system and cranial nerves [1–3]. Diagnosis of EG poisoning could be a challenge due to altered mental status and lack of poisoning history. While metabolic acidosis with increased anion gap is common, osmolar gap resolves within 24 to 72 hours as the EG is metabolized to toxic metabolites. Early initiation of treatment can reduce the mortality, and morbidity but different presentations especially with delayed cases could be a problem, so patients with acute renal failure require more attention [4].

In cases of renal failure with the suspicion of EG poisoning, kidney biopsy should be considered promptly. Histological examination of renal tissue often reveals widespread necrosis of the tubular epithelium and deposition of a multitude of doubly refractile oxalate crystals in the distal tubules and collecting ducts [5, 6].

Herein, we report a case with the atypical presentation of facial paralysis, hematuria, and kidney failure due to EG poisoning.

## 2. Case Report 

**Figure 1:**
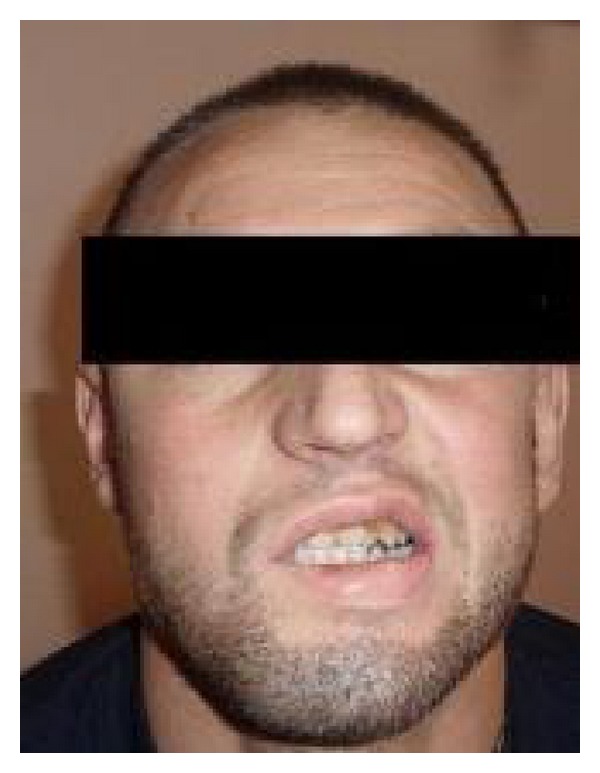
Unilateral right peripheral nerve palsy.

**Figure 2:**
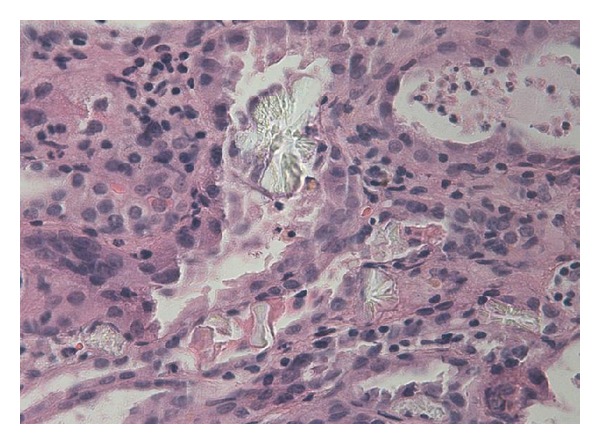
Light microscopy (H&E ×400) demonstrates deposition of doubly refractile oxalate crystals in the tubules.

**Figure 3:**
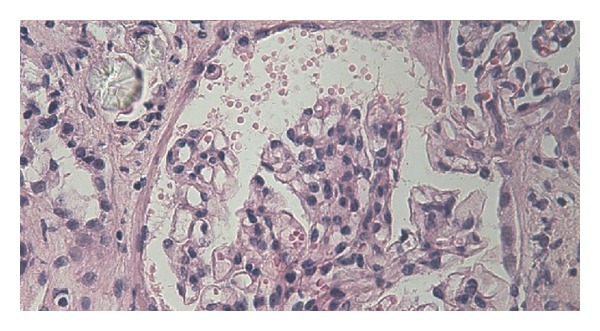
Light microscopy (H&E ×400) demonstrates oxalate crystal near glomerule.

A 25-year-old man was admitted to the emergency department of our university hospital with sudden onset of nausea, vomiting, slight mental disability, and abdominal pain. Cardiovascular, respiration, and gastrointestinal system examination findings were normal. The neurological examinations of the cranial nerves and limbs were normal. He was clinically dehydrated. On the first day of his admission, laboratory studies revealed a BUN level of 35 mg/dL, creatinine level of 3.17 mg/dL, calcium level of 8.6 mg/dL, and moderate metabolic acidosis with a pH of 7.30. The urinalysis showed hematuria [++++], leukocyturia [++], and proteinuria [+]. A lot of red blood cell casts and white blood cell casts with a few granular casts and epithelial cell casts were observed in urine sediment. Renal ultrasound showed normal sized kidneys with no hydronephrosis. The fractional excretion of sodium (FeNa) was 4%, and he was diagnosed with acute kidney failure. He was admitted to the nephrology department, and intravenous fluid therapy was started immediately due to his dehydration status. Kidney function tests and blood gases were monitored daily. His condition was stable until the 3rd day. On the 3rd day, anuria was developed, and his creatinine level increased to a level of 13,5 mg/dL and revealed a blood gas pH of 7.19 with high anion gap metabolic acidosis. He was admitted for urgent venovenous hemodialysis intervention for two hours via a jugular hemodialysis catheter. On the 4th day, a kidney biopsy was performed. On the 5th day, unilateral right peripheral nerve palsy developed abruptly (Figure 1). His temporal computerized tomography scan was normal, and electromyography showed a lesion on the right facial nerve. There was no history of cold exposure, and diabetes mellitus, vasculitis, and neuropathic disorders were ruled out by the negative autoantibody screen, normal serum electrophoresis, and negative HIV test. On the 7th, day pathological examination of the kidney specimen revealed tubular injury with oxalate crystals (Figures 2 and 3). The patient was questioned again after obtaining the pathological biopsy result. He then confessed that he had drunk antifreeze solution in a suicide attempt one day before being admitted to hospital. Hemodialysis intervention was continued three times a week due to progressive deterioration of kidney functions. He was discharged from hospital two weeks later with a hemodialysis catheter and followed up weekly. Two months later, he was considered as having end stage renal failure and underwent routine hemodialysis intervention via a created arteriovenous fistula. On the followup, the patient's facial palsy had not regressed.

## 3. Discussion

This case demonstrates the difficulties posed by hidden EG poisoning. In this case, our patient did not initially mention a history of toxin ingestion, and the precise diagnosis was delayed due to complicated clinical findings. It is possible that the moderate metabolic acidosis at presentation was due to the enhanced levels of glycolic acid and oxalic acid in the body. Our patient's presenting symptoms included nausea, vomiting, abdominal pain, and subsequently anuria and unilateral facial paralysis. However, the lack of the history of toxin ingestion and the development of anuria and unilateral facial nerve palsy pointed towards another diagnosis. 

The symptoms and signs of EG poisoning are traditionally divided into chronological stages. Initially, occurring within 12 hours of ingestion, the first stage consists of central nervous system depression and features of intoxication such as slurred speech and confusion. The second stage, occurring 12 to 24 hours after ingestion, is characterized by cardiovascular features and hyperventilation due to the production of acid metabolites. Eventually, the final stage occurs after 48 hours and is characterized by acute renal failure [7, 8]. There have been only a few case reports in the literature regarding EG poisoning causing delayed multiple cranial and peripheral neurologic deficits [8–10]. However, this neurological phenomenon is still not well described. Although patients with neurological symptoms would have previously died due to overdoses, with the improvements in medical care, these patients are now surviving and therefore demonstrating these delayed sequelae in recent years. These usually present about one to two weeks after the initial ingestion with cranial neuropathies, including bilateral facial palsy and ophthalmoplegia, as well as peripheral sensorimotor neuropathies which can be severe enough to cause complete paralysis [8, 11]. Patients with EG poisoning have shown both renal replacement requirement and recovery from acute renal failure, which does not seem to be associated with the degree of neurological recovery.

In the differential diagnosis, we considered other conditions including diabetes mellitus, vasculitis, rheumatoid arthritis, systemic lupus erythematosus, amyloidosis, sarcoidosis and HIV infection, which could also cause renal failure and neuropathies. These differentials were effectively ruled out with the negative autoantibody screen, normal serum electrophoresis, and negative HIV test and kidney biopsy.

In conclusion, delayed EG poisoning may involve different clinical presentations such as neurological symptoms as was so in our case. Physicians should be alert to patients with renal dysfunction and neurological findings even if there is no history of ingestion of EG.
